# The complete chloroplast genome sequence and phylogenetic analysis of Sudan grass (*Sorghum bicolor subsp. drummondii*) cultivar Sa (Poaceae) from Anhui province, China

**DOI:** 10.1080/23802359.2021.2001388

**Published:** 2021-11-23

**Authors:** Jieqin Li, Lihua Wang

**Affiliations:** College of Agriculture, Anhui Science and Technology University, Fengyang, China

**Keywords:** Chloroplast genome, phylogenetic tree, *Sorghum bicolor subsp. drummondii*, Sudan grass

## Abstract

The complete chloroplast genome of *Sorghum bicolor subsp. drummondii* cultivar Sa (a modern Sudan grass cultivar) was sequenced and analyzed in the present study. The chloroplast genome is 140,754 bp in length and includes a large single-copy region 82,688 bp in length, a small single-copy region 12,503 bp, and two inverted repeat regions 22,782 bp each. The genome contains 104 unique genes, including 4 rRNAs, 29 tRNAs, and 71 protein-coding genes. The phylogenetic analysis showed that Sudan grass cultivar Sa in a clade with five other complete chloroplast genomes of *S. bicolor*. The work facilitates studies on population genetic structure and phylogenetic relationships in genus *Sorghum*.

*Sorghum bicolor subsp. drummondii*, common name Sudan grass, is an important forage crop with remarkable drought tolerance (Creamer and Baldwin 2000). Sudan grass also has the potential to produce large amounts of biomass that builds soil quality in a short period of time. The plant also recycles nitrogen, outcompetes weeds, and reduces soil erosion (Acevedo et al. 2019). The cultivar Sa was widely planted in China because of its high biomass and excellent drought tolerance (Zhan et al. 2008). It is controversial about whether *Sorghum* and Sudan grass are the same species. Snowden (1936) treated Sudan grass as *S*. *sudanense*, a species different from *Sorghum* in spikelet, anthotaxy and plant traits. De Wet and Huckabay (1967) suggested that Sudan grass be placed as a subspecies, *drummondii*, of *S. bicolor* (L.) Moench. Zhan et al. (2008) also suggested that Sudan grass should be placed as a Sorghum subspecies under *S. bicolor* based on SSR markers evidence. In this study, we characterized the complete chloroplast genome of *Sorghum bicolor subsp. drummondii* Sa and explored its phylogenetic relationship within the genus *Sorghum*. The results will facilitate future studies on population genetic structure and phylogenetic relationships.

The Sudan grass cultivar Sa (voucher APGRCFC-S00001) was harvested from the planting station of Anhui Science and Technology University, Fengyang county (32°52′ N, 177°33′ E), Anhui province, China. The voucher specimen and its DNA were deposited at the Anhui Provincial germplasm resource center for forage crops (http://www.ahstu.edu.cn/nxy/info/1021/7873.htm, Jieqin Li, wlhljq@163.com). Total DNA was isolated from fresh leaves using the DNAsecure Plant Kit (Qiagen^TM^, Cat. No. DP320) following the manufacturer’s instructions. A library of circularized DNA fragments of 200–400 bp was amplified to make DNA nanoballs (DNBseq^TM^). Paired end 150 bp reads were generated on the MGISEQ-2000 sequencer. The raw data were filtered by SOAPnuke (Chen et al. 2018) with the filter parameters ‘-n 0.01 -l 20 -q 0.3 -A 0.25 –cutAdaptor -Q 2 -G –polyX 50 –minLen 150’. Then the complete chloroplast genome was assembled by GetOrganelle v1.7.4 (Jin et al. 2020). The genome sequences were annotated using GeSeq (Tillich et al. 2017) with the reference sequence of *Sorghum*. *bicolor* (NC_008602). The complete chloroplast genome sequence was submitted to NCBI under the accession number MW999225.

The complete chloroplast genome displayed the typical quadripartite structure found in most angiosperm chloroplast genomes (Luo et al. 2021), showing a high level of gene synteny to previously published *Sorghum* chloroplast genomes (Song et al. 2019). The genome includes a large single-copy (82,688 bp), a small single-copy (12,503 bp), and a pair of inverted repeats (22,782 bp). The GC content of chloroplast genome is 38%. The GC content for the large single-copy, the small single-copy, and the inverted repeats is 36%, 33%, and 44%, respectively. The genome encodes a total of 104 unique genes, of which there are 4 rRNAs s, 29 tRNAs and 71 protein-coding genes.

A phylogenetic analysis was performed based on complete chloroplast genome sequences from 16 *Sorghum* spp. and 1 *Sachharum* hybrid cultivar as the outgroup taxon. The 17 chloroplast genomes were aligned using MAFFT v7.313 with the auto settings (Katoh and Standley 2013). A Maximum likelihood phylogenetic tree was conducted using IQtree 1.68 (Nguyen et al. 2015) with the model K3Pu + F + I chosen according to the Bayesian analysis (BI) method and 1000 bootstrap replicates. Sudan grass cultivar Sa (*S. bicolor subsp. drummondii*) and *S. bicolor* were strongly resolved in the same clade (Figure 1). These results also suggested that Sudan grass should be classified as a subspecies in *S. bicolor* as Zhan et al. (2008) concluded using SSR markers.

**Figure 1.**
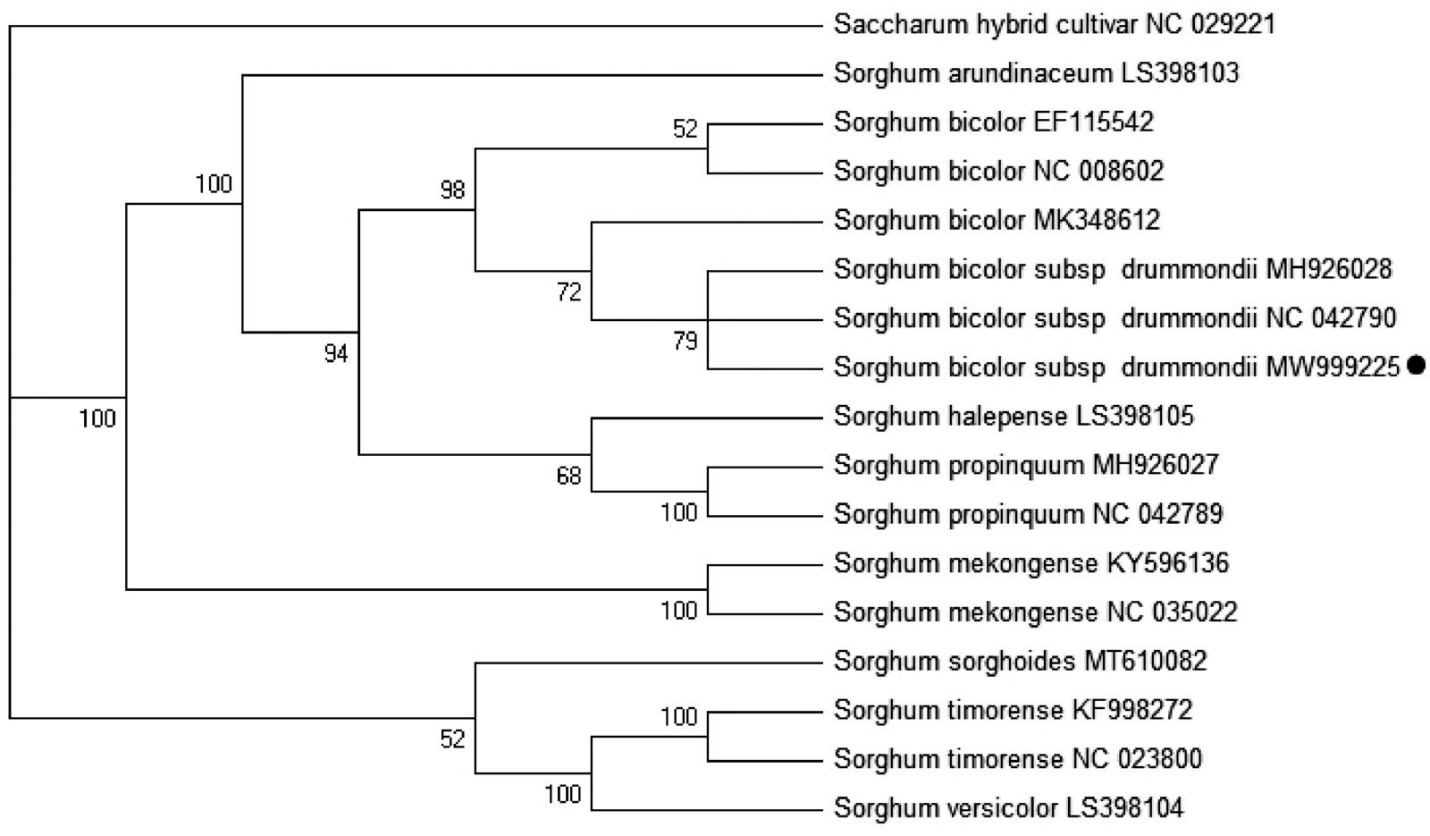
Maximum-likelihood tree based the 17 complete chloroplast genome sequences. The number at each node are the bootstrap percentages. The number after the species name is the GenBank accession number. The dark circle represents the chloroplast genome of the *Sorghum bicolor subsp.drummondii* cultivar Sa analyzed here in this study.

## Data Availability

The genome sequence data that support the results of the study are openly available in GenBank of NCBI at https://www.ncbi.nlm.nih.gov/ under the Accession no. MW999225. The associated BioProject and SRA are PRJNA718131 and SRR14089996, respectively. The Bio-sample number is APGRCFC-S00001in Anhui Provincial germplasm resource center for forage crops.
